# Revista de Saúde Pública: 50 years disseminating the knowledge in nutrition

**DOI:** 10.1590/S1518-8787.2016050000120

**Published:** 2016-11-24

**Authors:** Rosely Sichieri, Rosangela A Pereira

**Affiliations:** I Departamento de Epidemiologia. Instituto de Medicina Social. Universidade do Estado do Rio de Janeiro. Rio de Janeiro, RJ, Brasil; II Departamento de Nutrição Social e Aplicada. Instituto de Nutrição Josué de Castro. Universidade Federal do Rio de Janeiro. Rio de Janeiro, RJ, Brasil

**Keywords:** Diet, Food, and Nutrition, Nutrition, Public Health, Periodicals as Topic, Historical Article

## Abstract

This work describes and comments on articles in the area of Public Health Nutrition published in *Revista de Saúde Pública* (RSP – Public Health Journal) from 1967 to 2016. We searched in the PubMed database restricted to the periodical “Revista de Saúde Pública” and using terms related to key topics in the area of Public Health Nutrition. We retrieved 742 articles and, after exclusion of duplicates and articles unrelated to the subject, we analyzed 441 articles, grouped according to subject: dental caries, anemia, hypovitaminosis A, macro/micronutrients, malnutrition, nutritional assessment, overweight/obesity, food consumption, low birthweight, and breastfeeding. We observed significant increase in the number of articles published and diversification of subjects addressed over the 50 years, representing the consistent development of the scientific field of Nutrition in Brazil. Since its inception, RSP has played an important role in the dissemination of knowledge about the main nutritional issues in Brazil.

## RESUMO

Este trabalho descreve e comenta os artigos na área de Nutrição em Saúde Pública, publicados na Revista de Saúde Pública (RSP) de 1967 a 2016. Foi realizada busca na base de dados PubMed restrita ao periódico “Revista de Saúde Pública” e utilizando termos relacionados com temáticas chaves da área de Nutrição em Saúde Pública. Foram recuperados 742 artigos e, após as exclusões dos artigos repetidos e daqueles não relacionados com a temática, foram analisados 441 artigos, agrupados segundo o tema: cárie dental, anemia, hipovitaminose A, macro/micronutrientes, desnutrição, avaliação do estado nutricional, sobrepeso/obesidade, consumo de alimentos, baixo peso ao nascer, e aleitamento materno. Observou-se incremento significativo no número de artigos publicados e a diversificação dos temas tratados ao longo destes 50 anos, retratando o consistente desenvolvimento do campo científico da Nutrição no Brasil. Desde seu início, a RSP desempenhou importante papel na divulgação do conhecimento sobre os principais agravos nutricionais no Brasil.

## INTRODUCTION

At the time *Revista de saúde Pública* (RSP – Public Health Journal) was founded, the major problems of Public Health Nutrition in Brazil were anemia, protein-energy malnutrition, hypovitaminosis A, and iodine-deficient goiter^6^. This set also included dental caries, considered to date an important problem in public health. Dental caries is also an interesting study case in Nutrition. It is a historical case of ecological study on the association between nutrients available in the environment and dental caries, according to observations conducted in Colorado Springs, Colorado, United States, in the first decades of the 20th century, by Dr. Frederick S. McKay and revisited in a publication of the Centers of Diseases Control^a^.

In its history, RSP addressed the priority subjects of the agenda in Public Health Nutrition, reflecting the national researches conducted in the field, as Gandra^27^ demonstrated in a 1989 editorial. Briefly, the editorial emphasizes the populational approach as a source of important data from research in Nutrition in Brazil, providing well-founded information to national and state executive bodies, crucial to develop programs focused, for example: on surveillance of malnutrition, by monitoring children growth; on the interrelation between malnutrition and infection; on control of endemic goiter at national level and its surveillance system; on control of anemia and hypovitaminosis A in the country; and on the sociopsychomotor and mental development of malnourished children.

As the backdrop of the publications in the area of public health nutrition in Brazil, we highlight the importance of the research conducted by Josué de Castro, who, since the 1930s, presented a broad map of hunger and poverty in the Brazilian population, particularly the regional characteristics of food consumption and the occurrence of deficit in energy and nutrients intake^37^ ^,^ ^74^. In this context, also acquire importance, in the 1970s, the first National Food and Nutrition Programme (PRONAN) (established in 1972) and the first nationwide dietary survey in Brazil, the National Study of Family Expenditure (ENDEF)^b^. In the 1980s, several population-based studies carried out in different areas of Brazil, especially in the Northeast and in São Paulo, with an emphasis on protein-energy malnutrition were important bases for publications in RSP^17^ ^,^ ^48^, with emblematic articles involving the nutritional status assessment of children. Publications in the following decades reflect the changes in the epidemiological profile of Brazil, with the increment of researches involving diet as exposure factor for various outcomes.

This work describes and comments on articles in Public Health Nutrition published in the RSP since its foundation in 1967 to the present day, highlighting the important contribution of the journal for the dissemination of the scientific production in the area.

## METHODS

Search was performed in PubMed database (www.ncbi.nlm.nih.gov/pubmed) in June 2016, restricted to the periodical “Revista de Saúde Pública”, since its inception in 1967. We used the following terms (isolatedly): “*dental carie*”, “*anemia*”, “*A hypovitaminosis*”, “*malnutrition*”, “*protein energy malnutrition”*, “*undernutrition”*, “*stunting”*, “*nutrition assessment*”, “*nutritional status*”, “*anthropometry*”, “*obesity*”, “*overweight*”, “*diet*”, “*dietary assessment*”, “*dietary pattern”*, “*eating pattern*”, “*food consumption*”, “*dietary intake*”, “*calcium”*, “*vitamin A*”, “*vitamin D*”, “*vitamin C*”, “*riboflavin*”, “*folic acid*”, “*folate*”, “*thiamin*”, “*fat”*, “*birth weight*”, and “*breastfeeding*”. With these searches, 742 articles were retrieved. After exclusion of duplicates and of those not directly related to the subject (for example, articles related to dietary consumption of vectors and reservoirs), we analyzed 441 articles, which were classified into 10 subjects: dental caries, anemia, hypovitaminosis A, studies with macro/micronutrients, malnutrition, assessment of nutritional status, overweight/obesity, studies on food consumption, low birthweight , and breastfeeding.

We also estimated the proportion of articles published in RSP addressing the priority issues on the agenda of public health nutrition in comparison with total indexed records of such subjects in PubMed and Lilacs databases, using the following terms in English: *Protein-Energy malnutrition, Malnutrition, Dental Caries, Anemia, Vitamin A, Birth Weight, Breastfeeding*.

## RESULTS

**Figure 1:**
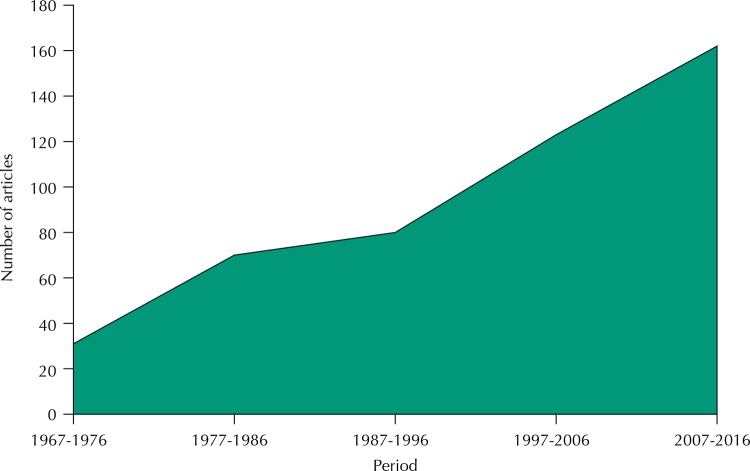
Articles in the area of Public Health Nutrition published in *Revista de Saúde Pública* according to the period (1967-2016).

Analysis of RSP publications in the area of public health nutrition showed significant increment of this subject over the years (Figure 1). In the first three years (1967-1969), only two articles addressed topics related to public health nutrition; in the last period assessed (2007-2016), more than 160 articles involving this subject were published. Even though having started with few articles on the area of public health nutrition, RSP excelled for the dissemination of knowledge about the main nutrition issues in Brazil, a characteristic that remains over time. Importantly, in the first publications most articles were by a single author and, from 1975, they started to be developed by multiple authors.

**Figure 2:**
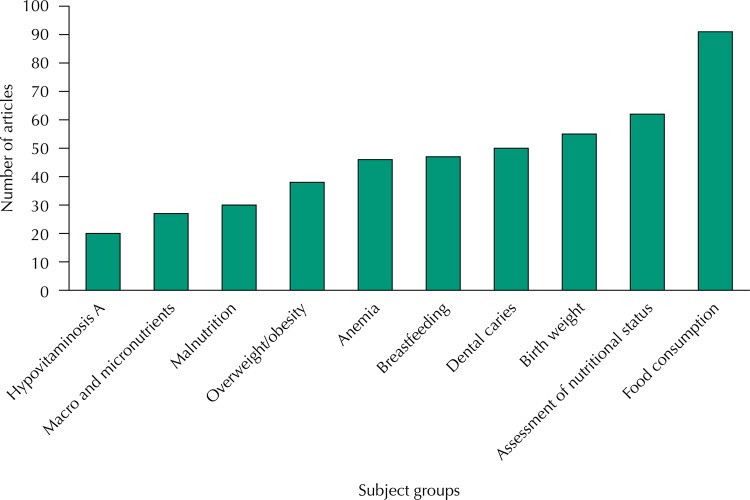
Number of articles in the area of Public Health Nutrition published in *Revista de Saúde Pública* (1967-2016) according to the subject.

The most relevant subjects addressed in the articles published in RSP on the area of public health nutrition include the historic public health problems mentioned above, as well as other issues that are fundamental to the public health agenda in Brazil, such as low birthweight, breastfeeding, and obesity. Noteworthy are the studies involving assessment of nutritional status and dietary intake (Figure 2).

**Figure 3:**
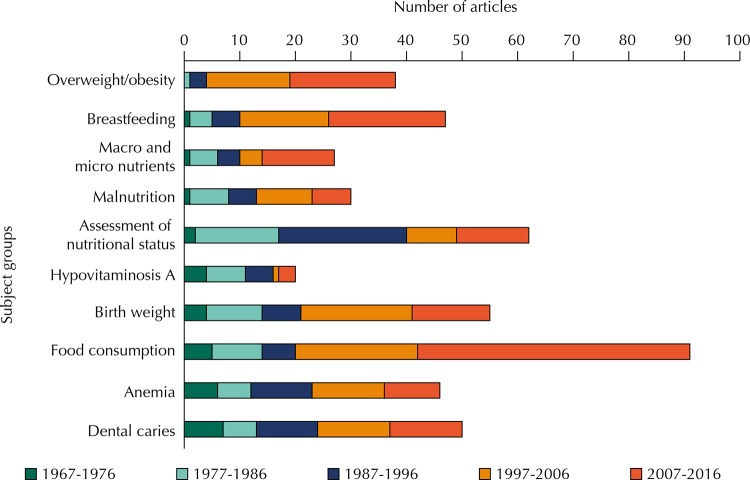
Number of articles in the area of Public Health Nutrition published in *Revista de Saúde Pública*, according to subject and publication period (1967-2016).

Analysis of the articles in the area of public health nutrition published in these 50 years of RSP shows that the subjects evaluated remained active throughout the period. It is remarkable the increase of publications addressing the evaluation of diet, especially from the mid-1990s. From this period, there is an increasing number of published articles on obesity. Interest in anemia remained stable over these years, while for malnutrition, vitamin A, and other micro and macronutrients interest was moderate (Figure 3). Most published articles refer to original research.

Studies on macro and micronutrients published in the journal were intended to diagnose nutritional deficiencies and assess methods used in evaluating the nutritional status of micronutrients, either using direct methods as biochemical analysis or estimating intake deficit based on food consumption studies. This subject was further explored in RSP in the 1970s and 1980s; however, from 1990 interest in the subject decreased. These analyses complemented both anthropometric assessment studies and food consumption studies. There were important publications on hypovitaminosis A, anemia, and dental caries, the latter treated as a result of fluoride deficiency.

In the first issue of RSP there is the first publication addressing a subject on Public Health Nutrition: dental caries^66^. The article presented results of investigation developed to evaluate differences in occurrence of dental caries according to skin color in low-income students. In the following years, nearly 60 articles (Figure 2) were published in RSP on this subject, including an investigation held in Brasilia in 1968, prior to measures of public water supply fluoridation, on the occurrence of dental caries in students^67^. Publications on dental caries remained over the 50 years in RSP, especially on the association of the disease with poverty, the availability of micronutrients (especially fluoride), methodological issues for diagnosis, and public policies. The latest publications are from 2013, when they published several studies on dental health in the different groups of the population^4^ ^,^ ^25^ ^,^ ^36^ ^,^ ^53^, its evolution in recent decades^52^, and its possible association with obesity^61^.

The subject hypovitaminosis A was addressed in RSP for the first time by Roncada^55^ in 1972 in an article that showed that this nutritional deficiency was a public health problem in the Vale do Ribeira region. This article was followed by 20 other on hypovitaminosis A, which additionally to registering the magnitude of the problem in different locations and population groups presented results of therapeutic tests^7^.

Anemia, regarded for many years as the public health problem of greater magnitude in Brazil, was initially addressed in article by Szarfarc^68^, also in 1972, describing the prevalence of the disease in the Vale do Ribeira region. Occurrence of anemia in different population groups, especially pregnant women and children, and its association with several outcomes – as children growth, result of gestation, and breastfeeding – were the subjects covered in about 50 publications of RSP (Figure 2).

From 1974 RSP published results of food surveys and their association with the nutritional status and other outcomes. Studies in this field of knowledge seek to assess the social factors associated with food consumption and methodological aspects dietary intake. The first articles deal with studies developed in the Vale do Ribeira region that applied the weighing method to obtain food consumption data, which showed deficit in intake of vitamin A, calcium, vitamin B_2_, and vitamin C^10^ ^,^ ^40^. In 2013, it published a supplement of articles presenting the results of the first nationwide nutrition survey, conducted in 2008–2009 along with the Family Expenditures Survey, developed by the Brazilian Institute of Geography and Statistics^3^ ^,^ ^5^ ^,^ ^8^ ^,^ ^18^ ^,^ ^22^ ^,^ ^65^ ^,^ ^75^. Food consumption studies was the area with the highest number of articles, about 20% of all articles analyzed in this work, with important growth from the mid-1990s (Figure 3). Some of the studies published on this subject are among the most cited among the articles analyzed here^33^.

Anthropometric assessment of nutritional status is a field of knowledge that is dedicated to develop and evaluate the tools used in the diagnosis of weight status in the various stages of life. Sampaio and Coelho^58^ published the first work on the subject of the assessment of nutritional status in RSP. Articles on this subject seek to discuss the methods and criteria adopted in the construction of indicators of nutritional status, identify social determinants and individual characteristics associated with the nutritional status, and evaluate the effect of interventions and programs. The subject evaluation of nutritional status was explored with more intensity in 1980–1990, but remains up to date, with recent articles published on the subject.

Initially, publications addressing the nutritional problems of the population highlighted malnutrition, subject of 30 articles published in RSP. Pioneering work^57^ found the impressive malnutrition rate of 75.0% in 2000 children under two years old admitted to a São Paulo municipal hospital in the 1960s. It is noteworthy a study published in 1974 that assessed the nutritional status of children enrolled in 23 schools located in slums, suburban neighborhoods, and housing estates intended for low-income families in the city of Londrina, PR. This study was the basis to establish the municipal assistance and nutrition education program^29^. Although it was not included in the articles analyzed in this study, one of the first publications in RSP already indicated the importance of malnutrition based on mortality analysis^41^. Works involving the subject malnutrition address various aspects, as anthropometric methods used to diagnose and classify the severity of protein-energy malnutrition^19^ ^,^ ^47^ ^,^ ^49^ ^,^ ^50^ and the association between protein-energy malnutrition and cognitive development and school performance^72^. In addition, the articles on protein-energy malnutrition clearly indicate the importance of social determinants in the onset of the disease^11^ ^,^ ^16^ ^,^ ^38^ ^,^ ^54^ and the country’s nutritional transition, which led to protein-energy malnutrition and nanism^45^ reduction and to overweight and obesity increase^28^ ^,^ ^31^ ^,^ ^34^ ^,^ ^43^.

The subject overweight and obesity appears initially in RSP in 1981 (Figure 3) in an article that evaluated the importance of obesity in gestation^69^, but would only reappear in RSP in 1991^35^ in an article that evaluated the prevalence of overweight and obesity in a location of the São Paulo state in 1987 and which showed high prevalence of overweight (about 27%). Five other articles on this subject were published between 1996 and 1999. Only from 2000 the subject obesity became recurring in RSP, representing about 8% of the evaluated publications and highlighting the importance that the issue of obesity acquires in the Brazilian epidemiologic context. Several articles emphasized the early development of obesity^9^ ^,^ ^12^ ^,^ ^15^ ^,^ ^21^ ^,^ ^24^ ^,^ ^30^ ^,^ ^51^ ^,^ ^64^ ^,^ ^70^ ^,^ ^71^,^79^. However, there are still few studies presenting results of interventions aimed at reducing overweight and obesity in children and adolescents^26^ ^,^ ^73^. The role of socioeconomic factors in the development of obesity was discussed in studies published by Alves and Faerstein^2^, while Veloso et al.^76^ and Chaparro et al.^14^ discussed the possible influence of public policies in nutrition over the occurrence of this disorder. As expected, the interrelation between obesity and the development of metabolic disorders was also the subject of works published in RSP^23^ ^,^ ^78^.

Breastfeeding and low birthweight were important issues of Brazilian public health agenda. They demanded actions to modify the unfavorable context that predominated in 1970-1980, when the prevalence of exclusive breastfeeding was extremely low^77^. These two subjects were a significant portion of the articles published in RSP on public health nutrition, beginning in the 1970s.

In 1973, Rosenburg^56^ denounces the gravity of the situation of breastfeeding in São Paulo, noting the “predominance of bottle-feeding over breastfeeding” and associating this to several factors, among them the “already established habit to do the free distribution of powdered milk in maternity wards and in different places of infant care.” The author points out that early weaning is aggravated by poor socioeconomic conditions, resulting in deficient diet and malnutrition^56^. As a result, nearly 50 articles were published in RSP addressing the topic of breastfeeding, assessing its prevalence, duration, associated factors, cultural aspects, impacts on health and nutrition, and evolution over the last few decades.

From the beginning, the articles published on low birthweight analyze biological and environmental factors associated with this outcome. Moreover, there are articles aiming at the analysis of intrauterine nutrition indicators^63^, while others investigate the effect of birthweight on health in childhood^20^ ^,^ ^59^ and adulthood^13^ ^,^ ^62^. Important analyses focused on the evolution of birthweight in the last decades^44^ and on those that highlight the paradox of low birthweight. This paradox concerns a phenomenon observed in several countries, with higher rates of low birthweight in developed areas compared to areas of poverty. According to Silva et al.^60^, a possible explanation would be the underregistration of live births in the less developed regions coupled with the increase of medical interventions in the more affluent populations. National data of live births over two decades show that the low birthweight paradox also occurs in Brazil^60^.

Until May 2016, RSP has published 4,146 records, out of which 4,048 were original articles. In the period of 50 years, the descriptor “protein-energy malnutrition” appears in 1.7% of publications indexed in Lilacs and in 0.3% of these publications in PubMed (Table). Thus, the assessment on publication of the subject in RSP is underestimated, since, until 1990, it already had 22 publications in the form of surveys in populations, hospitals, and kindergartens and schools or about diagnostic criteria for protein-energy malnutrition, higher number than the 21 retrieved with this descriptor (protein-energy malnutrition) over the 50 years of RSP. Furthermore, the table shows that other subjects often addressed in RSP relate to birthweight and malnutrition.

**Table:** Descriptors in Health Sciences by the Virtual Library of Medicine, general number retrieved and percentage in the Public Health Journal (RSP) according to LILACS and PubMed, May 2016.

Descriptor	Descriptor in English	RSP (n)	Lilacs			PubMed	
	
			n	% RSP		n	% RSP
Desnutrição Proteico-Calórica	Protein-Energy malnutrition	21	1,199		1.7	6.766	0.3
Desnutrição	Malnutrition	85	612		13.9	8.504	1.0
Cárie Dental	Dental Caries	73	3,458		2.1	35.262	0.2
Anemia	Anemia	59	1,259		4.7	30.581	0.2
Vitamina A	Vitamin A	28	551		5.1	17.773	0.2
Peso ao Nascer	Birth Weight	145	1,169		12.4	33.865	0.4
Aleitamento Materno	Breast Feeding	82	3,278		2.5	27.763	0.3

## DISCUSSION

Analysis of publications on public health nutrition in RSP, over its 50 years of existence (from 1967 to 2016), shows both increase in the number of publications involving this field of knowledge and diversification of topics covered. This evolution occurred consistently with the development of the scientific field of Nutrition in Brazil and in line with the public health priorities agenda. Over this period we can better know and understand the food and nutrition situation in Brazil based on articles published in RSP.

When RSP began his history, the most common nutritional deficiencies were protein-energy malnutrition, vitamin A deficiency, iron deficiency anemia, and iodine deficiency, detailed in publications of this periodical. Throughout this history, RSP has mirrored the significant transformations in the epidemiological context in Brazil, which coexists with the double nutritional burden, characterized by the superposition of deficiency diseases, still observed in significant portion of the population^39^, and by the growing presence of overweight in all regions of the country and for all groups of the population^c^.

The publications analyzed reflect the importance of the subjects in public health nutrition over these 50 years, as well as the implementation of population-based surveys conducted in large cities and in the country. Thus, the areas with greatest growth in publications were the evaluation of food consumption, the assessment of nutritional status, and obesity studies.

This prioritization regarding the subjects is also reflected in the statistics of citations in Scopus base (www.scopus.com) until September 2016. Of the three most cited articles among the articles assessed in this work, two address the temporal trends in food consumption in the country^33^ ^,^ ^46^, respectively with 206 and 109 citations, and the third, with 90 citations, analyzes the causes of the decline in child malnutrition in the country^43^.

The ENDEF (1974–1975)^b^ was the first investigation to represent effectively the nutrition situation in Brazil, obtaining anthropometric data and information on household food consumption. Since then, emphasis on anthropometric data in researches of national scope has enabled to assess the trend in the country’s nutritional condition, highlighting the changes in the population’s nutritional profile, the reduction of malnutrition, and the worrying increase of overweight. Moreover, the food survey developed in the ENDEF was a broad investigation on household food consumption, using the direct weighing method during one week^b^. This study was also related to family expenditure and enabled to observe what important part of household budget was spent on food; however, the diet of disadvantaged families was of low nutritional quality^1^.

Surveys on household expenditure with food have been conducted regularly since the 1970s, providing important information as for trends in the Brazilian dietary pattern, much properly evaluated in publications of RSP^32^ ^,^ ^33^ ^,^ ^42^ ^,^ ^46^, although with limitations inherent to the nature of the data. These works enable to infer that the Brazilian dietary pattern started to change between 1960 and 1970, with intensification of these changes in the 1980s and 1990s. According to Levy-Costa et al.^33^, food consumption in metropolitan areas was characterized by reduced consumption of staple foods, such as rice and beans, and increase in consumption of processed foods, especially soft drinks and cookies.

The first food survey with national representativity was developed only in 2008-2009. The data were published in RSP in a supplement^3^ ^,^ ^5^ ^,^ ^8^ ^,^ ^22^ ^,^ ^65^ ^,^ ^75^. This survey characterized the Brazilian diet of the early 21st century, showing that Brazilians had been combining the staple foods of good nutritional quality (such as rice and beans, which were the items most reported by the population) with foods of low nutritional quality and high energy density. It showed consumption below the recommended levels for fruits and vegetables and high intake of drinks with added sugar, particularly by teenagers. These characteristics result in high prevalence of dietary inadequacy compatible with increased rates of obesity and of chronic noncommunicable diseases, which mark the national context of morbidity and mortality. Finally, in its history, RSP reflected the important transformations that marked the country’s epidemiological context.

## Footnotes

a Centers for Disease Control and Prevention. Achievements in Public Health, 1990-1999: fluoridation of drinking water to prevent dental caries. *MMWR Morb Mortal Wly Rep*. 1999;48(41):933-40. Available from: https://www.cdc.gov/mmwr/preview/mmwrhtml/mm4841a1.htm

b Vasconcellos MTL, Ruchiga AE, Grilo CA. Metodologia do Estudo Nacional da Despesa Familiar- ENDEF: objetivos, descrição e metodologia usada no ENDEF. Rio de Janeiro: IBGE; 1983.

c Instituto Brasileiro de Geografia e Estatística. Pesquisa de Orçamentos Familiares 2008-2009: antropometria e estado nutricional de crianças, adolescentes e adultos no Brasil. Rio de Janeiro: IBGE; 2010.
